# Effects of overexpression of jasmonic acid biosynthesis genes on nicotine accumulation in tobacco

**DOI:** 10.1002/pld3.36

**Published:** 2018-01-25

**Authors:** Hongxia Chen, Bingwu Wang, Sisi Geng, Consuelo Arellano, Sixue Chen, Rongda Qu

**Affiliations:** ^1^ Department of Crop and Soil Sciences North Carolina State University Raleigh NC USA; ^2^ Yunnan Academy of Tobacco Agricultural Sciences Kunming China; ^3^ Department of Biology University of Florida Gainesville FL USA; ^4^ Department of Statistics North Carolina State University Raleigh NC USA

**Keywords:** jasmonic acid biosynthesis, jasmonic acid signaling, nicotine synthesis regulation, tobacco, transgene

## Abstract

Nicotine is naturally synthesized in tobacco roots and accumulates in leaves as a defense compound against herbivory attack. Nicotine biosynthesis pathway has been extensively studied with major genes and enzymes being isolated and functionally characterized. However, the molecular regulation of nicotine synthesis has not been fully understood. The phytohormone jasmonic acid (JA) mediates many aspects of plant defense responses including nicotine biosynthesis. In this study, five key genes (*AtLOX2*,* AtAOS*,* AtAOC2*,* AtOPR3*,* AtJAR1*) involved in JA biosynthesis from *Arabidopsis* were individually overexpressed, and a JA‐Ile hydrolysis‐related gene, *NtJIH1*, was suppressed by RNAi approach, to understand their effects on nicotine accumulation in tobacco. Interestingly, while transgene expression was high, levels of JA‐Ile (the biologically active form of JA) were often significantly reduced. Meanwhile, nicotine content in these transgenic plants did not increase. The research revealed a tightly controlled JA signaling pathway and a complicated regulatory network for nicotine biosynthesis by JA signaling.

## 1 INTRODUCTION

Tobacco *(Nicotiana tabacum* L.) is an important nonfood crop widely grown across the world due to its great economic value brought by the widespread usage of tobacco products (Davis & Nielsen, 1999). Nicotine is specifically synthesized in tobacco roots and accumulated in leaves as a defensive compound against herbivores because it causes a continual excitation of neurons and even paralysis or death of insects (Baldwin, Halitschke, Kessler, & Schittko, 2001). Thus, nicotine is used as an insecticide in agriculture practice (Davis & Nielsen, 1999). In medical applications, nicotine can be used to make smoking cessation devices (Wang et al., 2015) and is applied to treating Parkinson's disease and alleviating inflammatory bowel syndrome (Polosa, Rodu, Caponnetto, Maglia, & Raciti, 2013; Quik, O'Leary, & Tanner, 2008). Nicotine is a major type of alkaloids in tobacco plants, accounting for 90% of the total alkaloids. The rest 10% are mainly composed of anabasine, anatabine, and nornicotine (Saitoh, Nona, & Kawashima, 1985).

Nicotine is composed of a pyridine ring and a pyrrolidine ring, synthesized from two separate branches as demonstrated in Figure S1. The pyrrolidine ring is originated from arginine or ornithine while the pyridine ring is formed from quinolinic acid. Early studies reported that the putrescine methyltransferase (PMT) and quinolinic acid phosphoribosyltransferase 2 (QPT2) are the rate‐limiting enzymes in the pyrrolidine branch and pyridine branch, respectively, because they had much lower enzyme activities than other enzymes in the two branches (Feth, Wagner, & Wagner, 1986; Saunders & Bush, 1979; Wagner & Wagner, 1985). The isoflavone reductase‐like enzyme A622 and a berberine bridgelike (BBL) enzyme are proposed to be involved in the condensation step of the pyridine and pyrrolidine rings to form nicotine (DeBoer, Lye, Aitken, Su, & Hamill, 2009; Kajikawa, Hirai, & Hashimoto, 2009). The synthesis and accumulation of the major and minor alkaloids is closely related and dynamically regulated (Chintapakorn & Hamill, 2003; Hung et al., 2013; Kajikawa et al., 2009; Lewis et al., 2015).

Previous research indicates that many factors affect nicotine biosynthesis, including mechanical wounding, topping (decapitation of the apical meristem), plant hormones, transcription factors, and negative feedback by pathway products (Baldwin, Schmelz, & Ohnmeiss, 1994; Elliot, 1966; Wasternack & Hause, 2013). Topping and wounding induce nicotine biosynthesis through mediating phytohormones, mainly jasmonate (JA) and auxin. The transcription factor NtMYC2a is a master positive regulator for nicotine biosynthesis (Wang et al., 2015). Overexpression of *NtMYC2a* in tobacco plants increased nicotine content by approximately 1–1.5‐fold. RNAi‐induced knockdown of *NtMYC2* decreased the nicotine level by approximately fivefold (Wang, 2011). The high nicotine phenotype in *NtMYC2a* overexpression lines was consistent from T_0_ to T_3_ generations in field tests (Wang et al., 2015). It was shown that NtMYC2 upregulates nicotine biosynthesis by binding to the *cis* elements of an *NtPMT* and the *NtQPT2* promoter regions and activating the expression of these two genes (Zhang, Bokowiec, Rushton, Han, & Timko, 2012). Interestingly, previous research in our laboratory showed that overexpression of *NtPMT1a* and/or *NtQPT2* by a strong root‐specific promoter (*NtQPT2* gene promoter) did not change the nicotine content in a field‐grown commercial cultivar while the transcripts of these two genes increased, indicating a possible layer of regulation at post‐transcriptional levels (Wang, 2011). The speculation was recently confirmed by a miRNA–mimicry regulatory system on *QPT2* gene expression, and nicotine synthesis and accumulation (Li et al., 2015).

Jasmonic acid treatment induced expression of many genes involved in nicotine biosynthesis pathway and nicotine transportation (Baldwin et al., 1994; Goossens et al., 2003; Shoji & Hashimoto, 2011). Jasmonic acid regulates nicotine biosynthetic gene expression through the MYC2 and the jasmonate ZIM‐domain (JAZ) repressors system. In the absence of JA, the JAZs bind to MYC2 and form a repression complex, blocking MYC2 from activating nicotine biosynthetic genes. In the presence of JA, (+)‐7‐iso‐JA‐Ile forms a complex with COI1 and JAZs, releasing MYC2 transcription factor and activating nicotine biosynthesis (Kazan & Manners, 2013; Pauwels & Goossens, 2011; Wasternack & Hause, 2013). In addition, some ethylene responsive factors (ERFs) also play positive roles in nicotine biosynthesis (De Boer et al., 2011; Shoji & Hashimoto, 2011; Shoji, Kajikawa, & Hashimoto, 2010). Moreover, nicotine accumulation is regulated by a negative feedback loop as well: Nicotine itself is cellular toxic to tobacco root growth and negatively regulates its own biosynthesis (Shoji et al., 2009; Wang et al., 2015). Wang et al. (2015) demonstrated that transcript levels of all major nicotine synthesis genes in tobacco seedlings were reduced by about 50% 2 hr after 0.4 mM nicotine treatment, indicating a negative feedback pathway.

The JA biosynthesis pathway has been well established with major pathway components being functionally characterized, as shown in Figure S2 (Goossens, Fernández‐Calvo, Schweizer, & Goossens, 2016; Wasternack, 2007; Zhang, 2016). JA biosynthesis starts from α‐linolenic acid (C18:3), which is released from glycolipids of chloroplast membrane. The α‐linolenic acid is oxidized by lipoxygenases (LOXs) to form 13 (S)—hydroperoxy‐octadecatrienoic acid (13‐HPOT), which is then converted to 12,13 (S)‐epoxy‐octadecatrienoic acid [12,13 (S)‐EOT] by allene oxide synthase (AOS). The unstable allene oxide of 12,13 (S)‐EOT is immediately cyclized by allene oxide cyclase (AOC) to produce cis‐(+)‐12‐oxophytodienoic acid (OPDA). Thereafter, OPDA is translocated from the chloroplasts into the peroxisomes where the peroxisomal OPDA reductase (OPR) reduces OPDA. The reduction product goes through three rounds of beta‐oxidative side‐chain shortening to yield jasmonoyl‐CoA, which is then converted to (+)‐7‐*iso*‐JA. (+)‐7‐*iso*‐JA equilibrates to the more stable (−)‐JA, the predominant form of JA in plants. JA is released into the cytosol where it is metabolized to various derivatives, including JA‐Ile, the most active form of jasmonates. JA‐Ile formation is catalyzed by the JA amino acid synthetase (JAR1; Suza & Staswick, 2008).

Meanwhile, JA signaling could be attenuated by hydrolysis of JA‐Ile by jasmonoyl‐L‐isoleucine hydrolase 1 (JIH1; Woldemariam, Onkokesung, Baldwin, & Galis, 2012) and hydroxylation of JA‐Ile by p450 enzymes CYP94B3 and CYP94C1 (Miersch, Neumerkel, Dippe, Stenzel, & Wasternack, 2008). Although addition of methyl JA (MeJA) to the culture medium of tobacco BY‐2 cells has been shown to enhance alkaloids accumulation in the cells (Goossens et al., 2003), few reports studied effects on alkaloid levels by altering gene expression of JA metabolism pathways. Laudert, Schaller, and Weiler (2000) overexpressed *AtAOS* in tobacco but did not report its effects on nicotine accumulation. In another report, constitutive overexpression of the *Hyoscyamus niger* L. *AOC* gene in tobacco reportedly led to a notable fourfold to eightfold increase in *NtPMT* transcripts, a slight increase in *NtQPT2* transcripts, and a 4.8‐fold increase in nicotine content as compared to WT (wild type, Jiang et al., 2009).

In this study, we intended to gain more insights on JA regulated nicotine synthesis in tobacco by modifying expression of the JA biosynthetic genes. As we do not know which enzyme controls the rate‐limiting step of JA biosynthesis, we investigated all five major genes in the pathway. However, as none of the JA biosynthesis genes were cloned and functionally characterized in tobacco and the gene sequences were not available in the database at the time when we started this project, cDNAs of these genes from *Arabidopsis*,* LOX2, AOS, AOC2*,* OPR3*, and *JAR1,* were individually introduced into tobacco and constitutively overexpressed under the CaMV 35S promoter. In addition, RNAi‐mediated gene silencing was used to knock down *NtJIH1* (Woldemariam et al., 2012), which hydrolyzes JA‐Ile, in an attempt to enhance JA‐Ile level. The transgenic tobacco plants were developed with these individual gene constructs and analyzed.

## 2 MATERIALS AND METHODS

### 2.1 Generation of overexpression constructs

Vector pBI121 was used as the vector backbone and digested at *Bam*HI and *Eco*RI restriction sites. *Arabidopsis AOS* (AY128733), *AOC2* (AY054131), *OPR3* (AY097367), and *JAR1* (AY15043) cDNAs were acquired from Arabidopsis Biological Resource Center (ABRC, Ohio State University, Columbus, OH) while *LOX2* cDNA (L23968) was kindly provided by Dr. Carmen Castresana (Spain National Research Council, Madrid, Spain). The coding sequences of these genes were amplified by HiFi PCR premix (Clontech, Mountain View, CA), and the amplified sequences were verified by sequencing analysis. The amplified genes were inserted into the pBI121 to replace the *GUS* gene individually by homologous recombination using In‐Fusion HD cloning system (Clontech). Primers used for PCR are listed in Table S1.

### 2.2 Generation of *NtJIH1* RNAi construct

A Gateway binary RNAi vector pB7GWIWG2(II) (http://www.psb.ugent.be/gateway/) was used to generate inverted repeats and to express *NtJIH1* dsRNA in tobacco plants (McGinnis, 2010). Two copies of *NtJIH1* gene were found in tobacco genome database. A conserved 453‐bp region (Figure S3) was targeted by RNAi to knockdown both family members. The target region was amplified by HiFi PCR premix (Clontech) using sequence‐specific primers flanked by gateway attL sites (primers are shown in Table S1). PCR product was purified by ZymoClean gel DNA recovery kit (Zymo Research, Irvine, CA) and validated by sequencing analysis. An LR reaction was carried out to assemble the amplified fragment into pB7GWIWG2 (II) gateway vector using Gateway LR Clonase II Enzyme mix (Invitrogen, Carlsbad, CA).

### 2.3 Generation of transgenic plants

Transgenic tobacco plants were generated by *Agrobacterium‐*mediated leaf disk transformation as described (Horsch et al., 1989) with LBA4404 *Agrobacterium* strain harboring an individual gene construct. For overexpression constructs, the selection medium contained 100 mg/L kanamycin for *NPTII* gene selection. For the RNAi construct, the selection medium included 3 mg/L phosphinothricin for *bar* gene selection. After root formation, the plantlets were transplanted to greenhouse approximately 3 weeks later. PCR was performed on T_0_ plants to confirm the existence of the transgene(s) (primers used are listed in Table S1). PCR‐positive plants were grown to the preflowering stage and topped. Before topping, the third fully expanded leaves from the top were sampled for transgene expression analysis. Seven days after topping, the top 12 leaves from each plant were collected, dried, and mixed, and the total alkaloids levels were determined as an indicator of nicotine level. After sampling, flowers from suckers (branches developed from lateral buds after topping) of the T_0_ transgenic plants were self‐pollinated to produce T_1_ seeds.

### 2.4 Sampling of T_1_ plants for gene expression and alkaloids analysis

Two T_0_ plants per construct with higher transgene expression level and total alkaloids content were self‐pollinated to produce T_1_ generation plants. PCR was performed to identify transgenic plants in the T_1_ segregation population. Root tissues were pooled at preflowering stage from three plants to assess expression levels of nicotine synthesis‐related genes. Top 12 leaves of three T_1_ plants were collected 1 week after flowering in order to determine nicotine, nornicotine, anabasine, and anatabine contents.

### 2.5 Quantitative reverse transcription PCR (qRT‐PCR)

Leaf samples of T_0_ plants were collected for transgene expression. Root samples of T_1_ plants were collected for testing nicotine biosynthetic gene expression. Tissue samples were immediately frozen in liquid nitrogen after collection. Total RNA was isolated using the TRIzol Reagent (Invitrogen). RNA was treated with RQ1 DNase (Promega, Madison, WI) to remove genomic DNA. The cDNA was synthesized with iScript^™^ cDNA Synthesis Kit (Bio‐Rad, Hercules, CA). Real‐time qRT‐PCR was performed using the Stratagene Mx3005P system (Agilent Technologies, Santa Clara, CA) with iTaq^™^ Universal SYBR^®^ Green Supermix (Bio‐Rad). The thermal cycling program was set at 95°C for 30 s, followed by 40 cycles of 95°C for 5 s, and then 57°C for 30 s. Three technical repeats were performed for each assay. A tobacco *actin* gene (GenBank: U60490.1) was used as a reference gene for normalization. The gene‐specific primers are listed in Table S1.

### 2.6 Quantification of total alkaloids in T_0_ plants

Total alkaloids of T_0_ transgenic plants were determined by the segmented‐flow colorimetric method in the Tobacco Analytical Chemistry Laboratory at NCSU as previously described (Collins, Sarji, & Williams, 1969; Davis, 1976). In brief, the top 12 fully expanded leaves were collected from each tobacco plant 7 days after topping. Leaves were oven‐dried for 72 hr at 65°C and ground. Twenty‐five milliliter distilled water was added to 250 mg leaf sample. After 30 min of shaking, the aqueous extract was filtered through a Whatman filter paper. The collected aqueous extract was reacted with the sulphanilic acid buffer and cyanogen chloride buffer. The developed color was measured at 460 nm by colorimetry.

### 2.7 Quantification of individual alkaloids in T_1_ plants

At preflowering stage, the top 12 fully expanded leaves were collected and oven‐dried at 65°C for 72 hr and ground into fine powder. 0.2000 ± 0.0010 g of the leaf powder was weighed for total alkaloid extraction. Two milliliter 2 N NaOH solution was added to each sample. After incubation for 15 min, 10.0 ml quinoline working solution (0.4 g quinoline/ml methyl‐tert‐butyl ether) was added. After 2.5 hr of shaking, the mixture was incubated overnight for separation. Approximately 1.0 ml of the top methyl‐tert‐butyl ether (MTBE) layer was transferred into GC vials. Quantification was performed on an Agilent HP 6890 Gas Chromatograph (Agilent). One microliter sample from the GC vial was injected. The carrier gas helium was set at an average velocity of approximately 38 cm/s. The injector and detector were both set at 250°C. The oven temperature program was set as 110°C held for 1 min, 200°C at a rate of 10°C/min, 300°C at 25°C/min and held for 10 min. Data were collected and analyzed using Agilent Chemstation software. Pure nicotine (Sigma Aldrich, St. Louis, MO), anabasine (Alfa Aesar, Haverhill, MA), and nornicotine and anatabine (Toronto Research Chemicals, Toronto, Canada) were purchased and used as internal standards to make a calibration table.

### 2.8 Determination of JA‐Ile content in T_1_ lines

T_1_ lines were analyzed for JA‐Ile content, the biologically active form of JA. A 38‐mm‐diameter leaf disk was collected at the third fully expanded leaf from the top by a paper punch before flowering without topping/wounding treatment. The samples were immediately frozen in liquid nitrogen and stored in −80°C freezer until used. The frozen tissue was ground into fine powder, and 100 mg powder was quickly weighed before it thaws. One milliliter cold extraction buffer I (80% methanol spiked with 10 μl lidocaine as an internal control) was added. After well mixing, the mixture was vortexed at 4°C for 15 min, followed by sonication for another 15 min in ice water. After centrifugation at 15,700 *g* at 4°C for 15 min, the supernatant was transferred into a glass tube on ice. The extraction steps were repeated once more with 1 ml of cold extraction buffer II (acetonitrile: isopropanol: H_2_O = 3:3:2) and then with cold extraction buffer III (acetonitrile: H_2_O = 1:1). The supernatants from three extractions were combined in a glass tube. The total extracts were dried by a rotary evaporator at 30–40°C for approximately 15 min. The dry extracts were stored at −80°C until used. The residue was dissolved in 100 μl of sterile distilled water and centrifuged at 15,700 *g* at 4°C for 15 min, and the cleared supernatant was transferred to glass vials. HPLC‐MRM‐MS was performed using an Agilent 1100 HPLC (Agilent) coupled with an AB Sciex 4000 QTRAP^™^ (AB Sciex, Framingham, MA). Optimized detection conditions including precursor ion, product ion, declustering potential (DP), collision energy (EP), and cell exit potential (CXP) were established for quantification of JA, MeJA, JA‐Ile, OPDA. A reverse‐phase C18 column (Agilent, Eclipse XDB‐C18, 4.6 × 250 mm, 5 μm) was used for metabolite separation with 0.1% formic acid in water as solvent A and 0.1% formic acid in acetonitrile as solvent B. The LC gradient was as follows: 1% solvent B for 5 min; a linear gradient from 1% B to 99.5% B over 41.5 min; 99.5% B for 4.5 min; and return to 1% B. The flow rate was 0.5 ml/min, and the total analysis time was 1 hr. The mass spectrometer conditions were as follows: 30 psi curtain gas, 50 psi GS1, 55 psi GS2, ion source voltage at ±4,500 V, with the Turbo ElectroSpray Ionization (ESI) interface temperature at 350°C. A multiple period (segment) method was followed as previously described (Geng et al., 2016).

### 2.9 Statistical analysis

In nicotine content analysis of T_1_ lines, one‐way ANOVA and Dunnett comparison (α = .05) was used. For JA‐Ile content analysis, considering the relative heterogeneity within each genotype sample, a set of 1‐*df* contrasts was created in order to test the null hypothesis of transgenic JA‐Ile content is equal to control VC content, against the alternative hypothesis of VC having greater JA‐Ile content, at a significance level of .05. Each contrast analyzed the difference in JA‐Ile content between a particular genotype and VC, and the bootstrap stepdown method, for a one‐sided *t* test, was used within the SAS procedure MULTTEST (Satterthwaite analysis, SAS Institute Inc. 2015) to determine overall significance for this set of contrasts (Westfall & Young, 1993).

To improve the resolution of the statistical analysis, we conducted a follow‐up randomized complete block (RCB) design experiment in the greenhouse using two T_1_ lines with higher nicotine levels. PCR‐positive T_1_ plants of lines AOC2‐49, JAR1‐20, and WT were randomly assigned to three blocks with four plants in each plot. Plants were topped at preflowering stage, and leaves were collected 7 days after topping for nicotine analysis. Leaves from the same plot were pooled for nicotine analysis. The ANOVA and Tukey's studentized range test was used to determine difference significance and to make multiple comparisons.

## 3 RESULTS

Six to 11 T_0_‐independent transgenic plants were obtained from each gene construct. Plants transformed with the empty pBI121 vector (VC) or WT were used as a control. In T_0_ plants from two transgene constructs (*AtAOS* and *NtJIH1* RNAi), the transgene expression varied a great deal among T_0_ plants within a construct whereas the total alkaloids levels were comparable to, or even slightly lower than, VC (Figures S4 and S5), indicating very little effect of transgene expression on alkaloids synthesis. Thus, plants from these two constructs were not pursued further. However, some plants from the other four transgene constructs (*AtLOX2, AtAOC2, AtOPR3,* and *AtJAR1*) showed relatively higher total alkaloids contents at T_0_ generation. To further characterize these plants, two lines from each of these constructs were studied at T_1_ generation for expression of the five key nicotine synthesis‐related genes (*NtPMT1, NtQPT2, NtMYC2, NtBBLa,* and *Nt622*; Wang et al., 2015), and the contents of nicotine and the other three minor alkaloids (nornicotine, anabasine, and anatabine). Very small differences on the minor alkaloids were observed between transgenic plants and VC (Table S2). Results of gene expression and contents of total alkaloids or nicotine of these transgenic plants are reported below.

### 3.1 Overexpression of *AtAOS* or knockdown of *NtJIH1*

13‐AOS is the first dedicated enzyme in the JA biosynthesis pathway and catalyzes formation of unstable allene oxide (Wasternack, 2007). *Arabidopsis* has only one *AOS* gene in its genome (Kubigsteltig, Laudert, & Weiler, 1999). Six *AtAOS*‐overexpression T_0_ plants were generated. Various *AtAOS* transcript levels were detected in transgenic plants by qRT‐PCR (Figure S4a). The nicotine content of transgenic plants was similar to, or even slightly lower than, that of WT (Figure S4b), suggesting overexpression of *AOS* had little impact on nicotine biosynthesis.

Jasmonoyl‐L‐isoleucine hydrolase 1 (JIH1) catalyzes hydrolysis of jasmonoyl‐Ile, the active form of JA, so to regulate JA signaling in plants (Woldemariam et al., 2012). Seven *NtJIH1* RNAi plants were generated. Nicotine levels and *JIH1* transcript levels of these plants were determined. *JIH1* transcript levels were reduced in transgenic lines to only 8%–37% of that in WT (Figure S5a). However, nicotine content of transgenic plants was very similar to, or slightly lower than, WT (Figure S5b). These results suggest that *JIH1* downregulation does not affect nicotine synthesis.

### 3.2 Overexpression of *AtLOX2*

**Figure 1:**
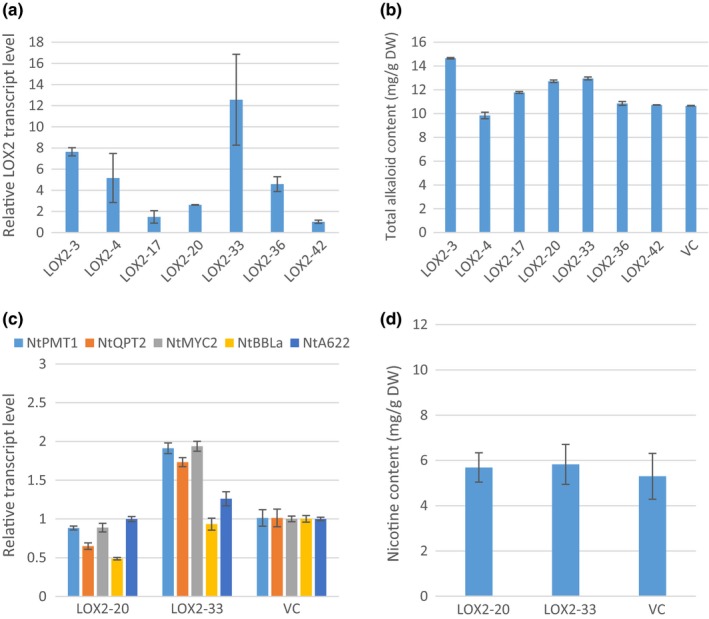
Overexpression of AtLOX2 in tobacco. (a) Relative AtLOX2 transcript levels in T_0_ plants RNA were isolated from leaf tissues collected before topping. Transcript levels were determined by qRT‐PCR. Values are means from three technical replicates. AtLOX2 transcript levels are normalized to actin and presented as fold to the AtLOX2 level in LOX2‐42 plant which is arbitrarily set at 1. (b) Total alkaloid levels of T_0_ plants. Total alkaloids (mg/g dry weight) were extracted and quantified from leaves 7 days after topping. The values shown are the means of total alkaloids (mg) per gram leaf dry weight from three technical replicates. (c) Transcript levels of five nicotine synthesis‐related genes in roots of two T_1_ lines. Roots from three plants were pooled at preflowering stage. Transcript levels were determined by qRT‐PCR with three technical replicates. Transcript levels were normalized to actin and presented as fold to the vector control (VC) which was arbitrarily set at 1. (d) Nicotine content of two T_1_ lines. Nicotine was extracted and quantified from leaves at preflowering stage. The values shown are the means of nicotine (mg) per gram of leaf dry weight from three biological replicates. VC, vector control. No significant difference was observed

13‐Lipoxygenase (13‐LOX) catalyzes oxygenation of α‐linolenic acid at an early step of JA biosynthesis. Among four *13‐LOX* genes in *Arabidopsis*,* LOX2* is responsible for the bulk JA formation at early wounding, and generation of oxylipins (Wasternack & Hause, 2013). Seven independent transgenic plants were obtained. The qRT‐PCR results indicate that all the transgenic plants had detectable *AtLOX2* transcript levels (Figure 1a). The total alkaloid increase ranges from 8% to 47% (Figure 1b). Two *LOX2* overexpression plants (LOX2‐20 and LOX2‐33) were chosen for further analysis at the T_1_ generation. *NtPMT1*,* NtQPT2*,* NtMYC2*,* NtBBLa,* and *NtA622* transcript levels in LOX2‐20 T_1_ line were comparable to, or slightly lower than, those in VC. In LOX2‐33 line, however, the *NtPMT1*,* NtQPT2*, and *NtMYC2* transcripts were doubled as compared to VC (Figure 1c). The means of nicotine content of the two transgenic lines were slightly higher than those of VC (Figure 1d); however, the difference was not statistically significant.

### 3.3 Overexpression of *AtAOC2*

**Figure 2:**
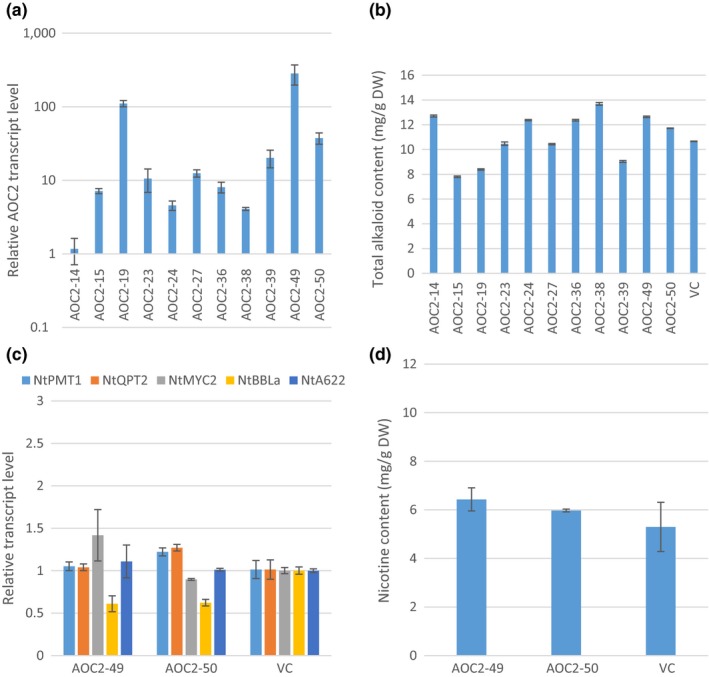
Overexpression of AtAOC2 in tobacco. (a) Relative AtAOC2 transcript levels in T_0_ plants. RNA was isolated from leaf tissues collected before topping. Transcript levels were determined by qRT‐PCR. Values are means from three technical replicates. AtAOC2 transcript levels were normalized to actin and presented as fold to the AtAOC2 level in AOC2‐14 plant which is arbitrarily set at 1. (b) Total alkaloid levels of AOC2 T_0_ plants. Total alkaloids were extracted and quantified from leaves 7 days after topping. The values shown are the means of total alkaloids (mg) per gram leaf dry weight from three technical replicates. (c) Transcript levels of five nicotine synthesis‐related genes in roots of two AOC2 T_1_ lines. Roots from three plants were pooled at preflowering stage. Transcript levels were determined by qRT‐PCR with three technical replicates. Transcript levels were normalized to actin and presented as fold to the vector control (VC) which was arbitrarily set at 1. (d) Nicotine content of two AOC2 T_1_ lines. Nicotine was extracted and quantified from leaves at preflowering stage. The values shown are the means of nicotine (mg) per gram of leaf dry weight from three biological replicates. VC, vector control. No significant difference was observed

There are four allene oxide cyclase (AOC) isoforms with partial functional redundancy in *Arabidopsis*, among which function of AOC2 is better understood (Wasternack & Hause, 2013). Among 11 *AtAOC2* overexpression T_0_ plants, the transgene expression varied by hundreds of fold (Figure 2a). However, total alkaloids contents varied only from 26% reduction up to 19% increase as compared to VC (Figure 2b). Lines AOC2‐49 and AOC2‐50 were chosen for further analysis at T_1_ generation. The basal levels of *NtPMT1, NtQPT2, NtMYC2, NtBBLa,* and *NtA622* transcripts in these transgenic plants were similar to VC (Figure 2c). The means of nicotine content in the AOC2‐49 and AOC2‐50 transgenic plants were 10%–20% higher than VC (Figure 2d). However, the differences were not statistically significant.

### 3.4 Overexpression of *AtOPR3*

**Figure 3:**
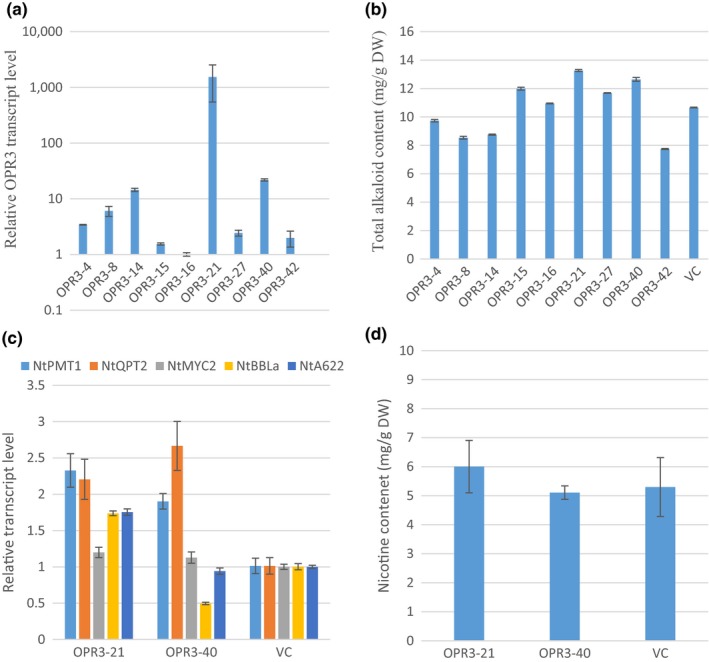
Overexpression of AtOPR3 in tobacco. (a) Relative AtOPR3 transcript levels in OPR3 T_0_ transgenic plants. RNA was isolated from leaf tissues collected before topping. Transcript levels were determined by qRT‐PCR. Values are means from three technical replicates. AtOPR3 transcript levels were normalized to actin and presented as fold to the AtOPR3 level in OPR3‐16 plant which is arbitrarily set at 1. (b) Total alkaloid levels of OPR3 T_0_ plants. Total alkaloids were extracted and quantified from leaves 7 days after topping. The values shown are the means of total alkaloids (mg) per gram leaf dry weight from three technical replicates. (c) Transcript levels of five nicotine synthesis‐related genes in roots of two OPR3 T_1_ lines. Roots from three plants were pooled at preflowering stage. Transcript levels were determined by qRT‐PCR with three technical replicates. Transcript levels were normalized to actin and presented as fold to the vector control (VC) which was arbitrarily set at 1. (d) Nicotine content of two OPR3 T_1_ lines. Nicotine was extracted and quantified from leaves at preflowering stage. The values shown are the means of nicotine (mg) per gram of leaf dry weight from three biological replicates. VC, vector control. No significant difference was observed

Among six OPDA reductases (OPR) in *Arabidopsis*, only OPR3 is involved in JA biosynthesis (Wasternack & Hause, 2013). Nine transgenic plants overexpressing *AtOPR3* were generated. The transgene expression varied by more than 1,000‐fold (Figure 3a), and four T_0_ plants had total alkaloid content increase, ranging from 14% to 43% as compared to VC (Figure 3b). Two transgenic lines, OPR3‐21 and OPR3‐40, were chosen to be further studied at T_1_ generation.

In T_1_ generation of both lines, *NtPMT1* and *NtQPT2* transcript levels were doubled of those in VC (Figure 3c). In OPR3‐21 line, *NtBBLa* and *NtA622* transcript levels had more than 50% increase as compared to VC. However, the means of nicotine content in transgenic plants are not significantly different from VC (Figure 3d). Based on these data, we conclude that overexpression of *OPR3* somewhat enhanced nicotine pathway gene transcript levels, but had little effect on nicotine accumulation.

### 3.5 Overexpression of *AtJAR1*

**Figure 4:**
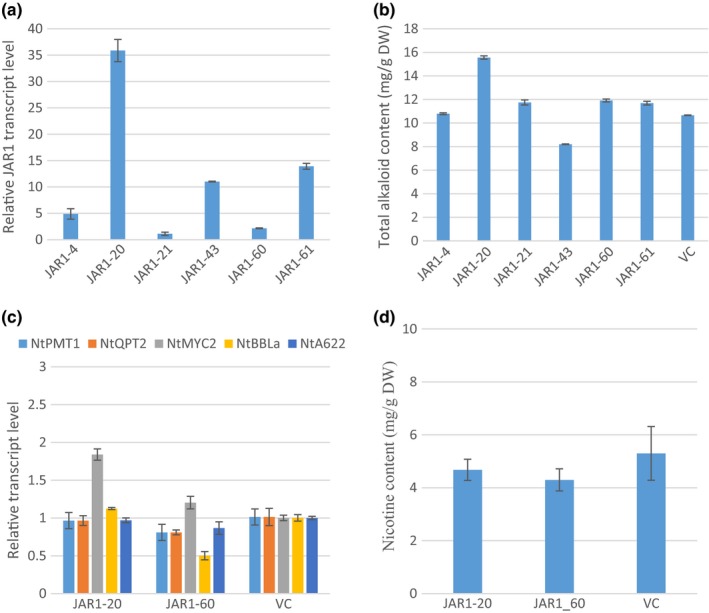
Overexpression of AtJAR1 in tobacco. (a) Relative AtJAR1 transcript levels in JAR1 T_0_ transgenic plants. RNA was isolated from leaf tissues collected before topping. Transcript levels were determined by qRT‐PCR. Values are means from three technical replicates. AtJAR1 transcript levels were normalized to actin and presented as fold to the AtJAR1 level in JAR1‐21 plant which is arbitrarily set at 1. (b) Total alkaloid levels of JAR1 T_0_ plants. Total alkaloids were extracted and quantified from leaves 7 days after topping. The values shown are the means of total alkaloids (mg) per gram leaf dry weight from three technical replicates. (c) Transcript levels of five nicotine synthesis‐related genes in roots of two JAR1 T_1_ lines. Roots from three plants were pooled at pre‐flowering stage. Transcript levels were determined by qRT‐PCR with three technical replicates. Transcript levels were normalized to actin and presented as fold to the vector control (VC) which was arbitrarily set at 1. (d) Nicotine content of two JAR1 T_1_ lines. Nicotine was extracted and quantified from leaves at pre‐flowering stage. The values shown are the means of nicotine (mg) per gram of leaf dry weight from three biological replicates. VC, vector control. No significant difference was observed

Six *AtJAR1* transgenic plants were generated. More than 30‐fold difference of *AtJAR1* transcript level was detected among the six plants (Figure 4a). Five of them had increased total alkaloid content as compared to VC (Figure 4b). For example, in JAR1‐20 and JAR1‐60 plants, total alkaloid increased by 50% and 11.7%, respectively. When the two lines were further analyzed at T_1_ generation, compared to VC, the basal levels of *NtPMT1*,* NtQPT2*,* NtBBLa,* and *NtA622* transcript did not change, but *MYC2* was nearly doubled in JAR1‐20 and almost unchanged in JAR1‐60 (Figure 4c). However, no significant changes in the nicotine levels were detected, as shown in Figure 4d.

### 3.6 An effort to improve statistical analysis

Due to elevated total alkaloid content in the T_0_ generation (Figures 2 and 4), some lines, such as AOC2‐49 and JAR1‐20, looked more likely to have elevated nicotine content (Saitoh et al., 1985), and the sensitivity of the statistical analysis in the T_1_ experiments might have been suffered by the limited sample size. To improve the resolution of the statistical analysis, we conducted a follow‐up randomized complete block (RCB) design experiment in the greenhouse. Such design could help control location effect and reduce variance among the replicates. However, the calculated minimum significant difference is 3.7454, indicating that the nicotine levels of lines AOC2‐49 and JAR1‐20 were not significantly different from WT (Table 1).

**Table 1:** Nicotine levels of T_1_ plants of lines AOC2‐49 and JAR1‐20 after topping in a randomized complete block design experiment. (A) Analysis of variance of the RCB experiment. PCR‐positive T_1_ plants of lines AOC2‐49 and JAR1‐20, and WT were randomly assigned to three blocks in the greenhouse. Each plot contained four plants of a line. The third leaves from the top of each plot were pooled 7 days after topping, and nicotine levels were determined by GC‐MS. (B) Tukey's studentized range test of nicotine level in the RCB experiment shows no significant differences between transgenic and WT lines

A					
Source of variation	*df*	Sum of squares	Mean square	*F* Value	Pr > *F*
Model	5	60.28889672	12.05777934	2.27	.1275
Error	6	26.59059696	4.43176616		
Location	3	32.12215774	10.70738591	2.42	.1648
Treatment effect	2	28.16673898	14.08336949	3.18	.1145
Corrected total	11	86.87949368			

B			
Tukey grouping	Mean (mg/g DW)	*N*	Treatment
A	9.676	4	AOC2_49
A	6.564	4	JAR1_20
A	6.303	4	WT

### 3.7 Overexpressing JA synthesis pathway genes did not increase JA‐Ile content

**Figure 5:**
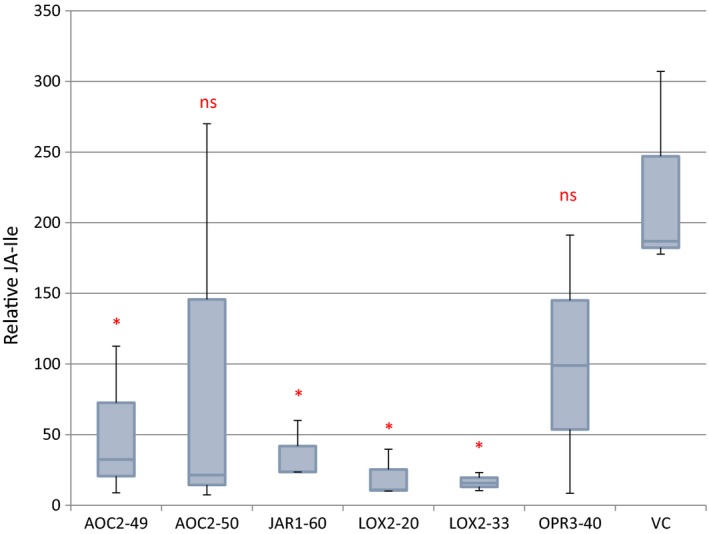
JA‐Ile contents in non‐topped transgenic T_1_ lines, and VC. 0.1 g of the third fully expanded leaves from top was collected from PCR positive transgenic plants (n = 3) of T_1_ lines prior to flowering, and the JA‐Ile peak area was measured. Bootstrap stepdown method is used to test the null hypothesis that genotype does not differ significantly from the control VC, with a one‐sided t test and the SAS procedure MULTTEST (Satterthwaite analysis) to determine significance. The x‐axis value (×10^4^) represents JA‐Ile peak area per gram tissue normalized to internal control on the HPLC graph. *Significant difference at α = .05. ns, not significant

Among JA and its derivatives in plants, JA‐Ile is the bioactive form functioning in JA signaling and regulation of downstream genes. To further understand how overexpression of JA synthesis pathway genes affects nicotine biosynthesis, JA‐Ile contents of six T_1_ lines with various transgene constructs were analyzed. As shown in Figure 5, four lines (AOC2‐49, JAR1‐60, LOX2‐20, and LOX2‐33) of six had significant JA‐Ile reduction (*p* < .05) when their means were compared with VC. The other two lines (AOC2‐50 and OPR3‐40) also had a great reduction in JA‐Ile. Obviously, the JA‐Ile contents in transgenic plants were substantially reduced by overexpression of the transgenes from the JA synthesis pathway.

## 4 DISCUSSION

In this research, five *Arabidopsis* JA synthetic genes, *LOX2, AOS, AOC2, OPR3,* and *JAR1,* were individually introduced into tobacco genome and constitutively overexpressed. The *NtJIH1* gene in tobacco, which hydrolyzes JA‐Ile, was knocked down by RNAi‐mediated gene silencing with an intention to reduce the degradation of JA‐Ile. The most striking phenomenon we observed in this experiment is that overexpression of these JA biosynthesis genes did not increase, and often reduced, JA‐Ile contents in transgenic plants. Laudert et al. (2000) recorded similar results: When *AtAOS* was overexpressed in tobacco, the basal JA level was reduced by nearly 10‐fold. The authors argued that the supply of the upstream substrate of JA synthesis pathway, α‐linolenic acid, is limited without wounding (Laudert et al., 2000). Although it could explain no increase in JA‐Ile, it cannot explain substantial reduction in JA‐Ile in the transgenic plants (their result and our Figure 5). Our data suggest that regulation of JA signaling is complicatedly regulated and tightly controlled at the various steps of JA biosynthesis. Because defense signaling is resource‐costly, plant fine‐tunes its responses to attenuate JA signaling at various levels. For instance, it is well documented that JA signaling negatively affects root cell division and thus root length (Wasternack & Hause, 2013). To tightly regulate JA signaling, plant developed fine tuning and JA attenuation mechanisms at various levels. For example, when studying the crystal structure of tomato OPR3, the authors unexpectedly observed a self‐inhibited dimer structure (Breithaupt et al., 2006), suggesting a strong and reversible dimerization in vivo involving phosphorylation of OPR3 as a regulatory step in JA biosynthesis. In another case, it was observed that any two of the four functional AOC polypeptides, AOC1, 2, 3, and 4, were able to interact to each other in BiFC experiments, prompting the authors to propose another layer of regulatory mechanism in JA biosynthesis through various combinations of heterodimerization of the four AOCs (Stenzel et al. 2012).

The second striking phenomenon we observed is that no matter how expression of nicotine synthesis‐related genes changed (or unchanged), the nicotine contents roughly stayed the same as in VC (Figures 1, 2, 3, 4c,d). The situation is similar to a previous observation in our laboratory: Overexpression of *NtPMT1a* and/or *NtQPT2* under the *NtQPT2* promoter did increase the transcript levels of both genes, but did not alter the nicotine content in the field test (Wang, 2011). However, knocking down expression of these genes by antisense or co‐suppression did reduce nicotine contents (Chintapakorn & Hamill, 2003; Wang, 2011; Xie et al., 2004), implicating that these key nicotine synthesis pathway genes are necessary but not sufficient in nicotine synthesis and accumulation. Based on these observations, we propose that nicotine biosynthesis in tobacco is also tightly regulated at multiple levels. One such a regulatory step is at transcriptional level. As shown previously, overexpression of transcription factors NtMYC2a or NtERF189 and alike could enhance expression of nicotine synthesis pathway genes leading to higher nicotine content in tobacco plants (Wang et al., 2015) or cultured cells (De Boer et al., 2011; Shoji & Hashimoto, 2011; Shoji et al., 2010). In addition, a negative feedback regulation was reported at the transcriptional level of *AtMYC2*, which is a “master” TF at the center of JA signaling (Kazan & Manners, 2013) and positively regulates pathway gene expression of nicotine synthesis (Wang et al., 2015). It was shown that *AtMYC2* promoter has an MYC2 binding site, and thus, *MYC2* was able to directly, negatively regulate its own transcript level (Dombrecht et al. 2007).

Furthermore, post‐transcriptional regulation is also shown to play an important role in nicotine biosynthesis. One example is the recently revealed mimicry and microRNA system tobacco uses to regulate the transcript level of *NtQPT2* gene and thus the nicotine biosynthesis (Li et al., 2015). The nta‐eTMX27, the mimicry of *NtQPT2* transcript, is upregulated after topping while the corresponding microRNA, nta‐miRX27, is downregulated, and the nicotine content increases. Moreover, phosphorylation of an MYC2 (NbbHLH1) and/or an ERF by an MAPKK (NtJAM1) further enhances the activities of these TFs and nicotine accumulation (De Boer et al., 2011), indicating a regulation step at post‐translational level. All these suggest that, in future experiments to study nicotine synthesis regulation, the enzymatic activities of NtPMT and NtQPT2, rather than their mRNA levels, need to be measured in both transgenic plants and control plants. Additionally, nicotine itself has a negative feedback loop on expression of its synthesis pathway genes (Wang et al., 2015) although the detailed mechanism(s) still needs to be elucidated.

Another interesting observation in our research is that reduction at JA‐Ile level did not affect nicotine contents (Figures 1, 2, 3, 4d), seemingly to suggest that the basal nicotine level (without wounding/topping) in tobacco be regulated by a pathway other than JA signaling. Alternatively, the endogenous JA‐Ile level may not correlate well with the expression of the JA signaling pathway genes and the accumulation of the nicotine content due to the complicated regulatory mechanisms in plant defense responses (De Vos et al., 2005).

In addition, we observed that total alkaloids contents measured in T_0_ generation often varied a great range, probably more sensitive to environmental factors, and did not appear to be reliable. Conversely, nicotine content in T_1_ generation looks more stable although we do not have a good explanation for the phenomenon.

Unlike the observations in our study, a previous research reported that constitutive overexpression of an *AOC* gene from a Solanaceae species, *Hyoscyamus niger* L., led to a 4.8‐fold increase in nicotine yield in tobacco plants (Jiang et al., 2009). The inconsistency may be caused by two possible reasons. First, the AOC from *Hyoscyamus niger* L. may have higher enzyme activity and thus stronger JA signaling effects. When the HnAOC (248 AAs) and AtAOC2 (253 AAs) proteins are analyzed, only 175 AAs can be aligned and 65% of those are identical. It is likely that the AOC enzyme activities from these two species are different. Second, Jiang et al. seem to have analyzed T_0_ transgenic plants only, and our data indicated that alkaloids data from T_0_ generation are not quite reliable (Figures 1, 2, 3, 4).

Taken together, overexpression of the key genes in JA biosynthesis pathway or RNAi approach of a catabolic gene is not an efficient way to alter nicotine biosynthesis, which is repeatedly shown in our experiments. The research revealed a tightly controlled JA signaling pathway and a very complicated regulatory network for nicotine biosynthesis.

## AUTHOR CONTRIBUTIONS

HC performed most of the experiments and collected the data. HC, BW, and RQ designed the experiments, analyzed the data, and wrote the manuscript. SG and SC analyzed the content of JA‐Ile and discussed the data. CA and HC performed statistical analysis of the data. All authors read and approved the manuscript.
