# Supplementary material for: Effects of overexpression of jasmonic acid biosynthesis genes on nicotine accumulation in tobacco
Source: Plant Direct. 2018 Jan 25;2(1):e00036. doi: 10.1002/pld3.36 (PMC6508566; doi:10.1002/pld3.36)
Supplement: Supplementary file 1 [file PLD3-2-e00036-s001.pdf]

Supplemental Materials

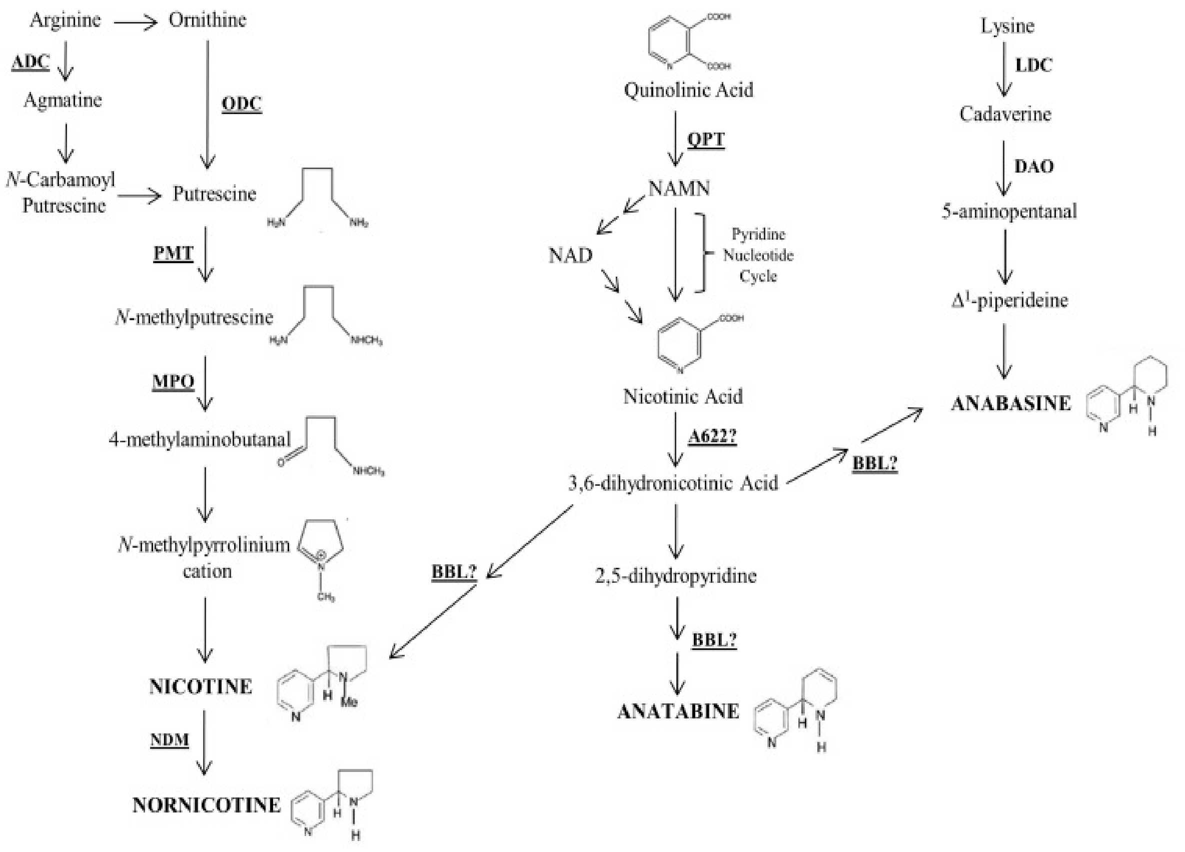

Figure S1. Scheme of Nicotine biosynthesis pathway

Adopted from Lewis et al. (2015). ADC, arginine decarboxylase; ODC, ornithine decarboxylase; PMT, putrescine methyltransferase; MPO, N-methylputrescine oxidase; QPT, quinolinate phosphoribosyltransferase; A622, isoflavone reductase-like protein; BBL, berberine bridge enzyme-like; DAO, diamine oxidase; LDC, lysine decarboxylase; NDM, nicotine demethylase; NAMN, niacin mononucleotide; NAD, nicotinamide adenine dinucleotide.

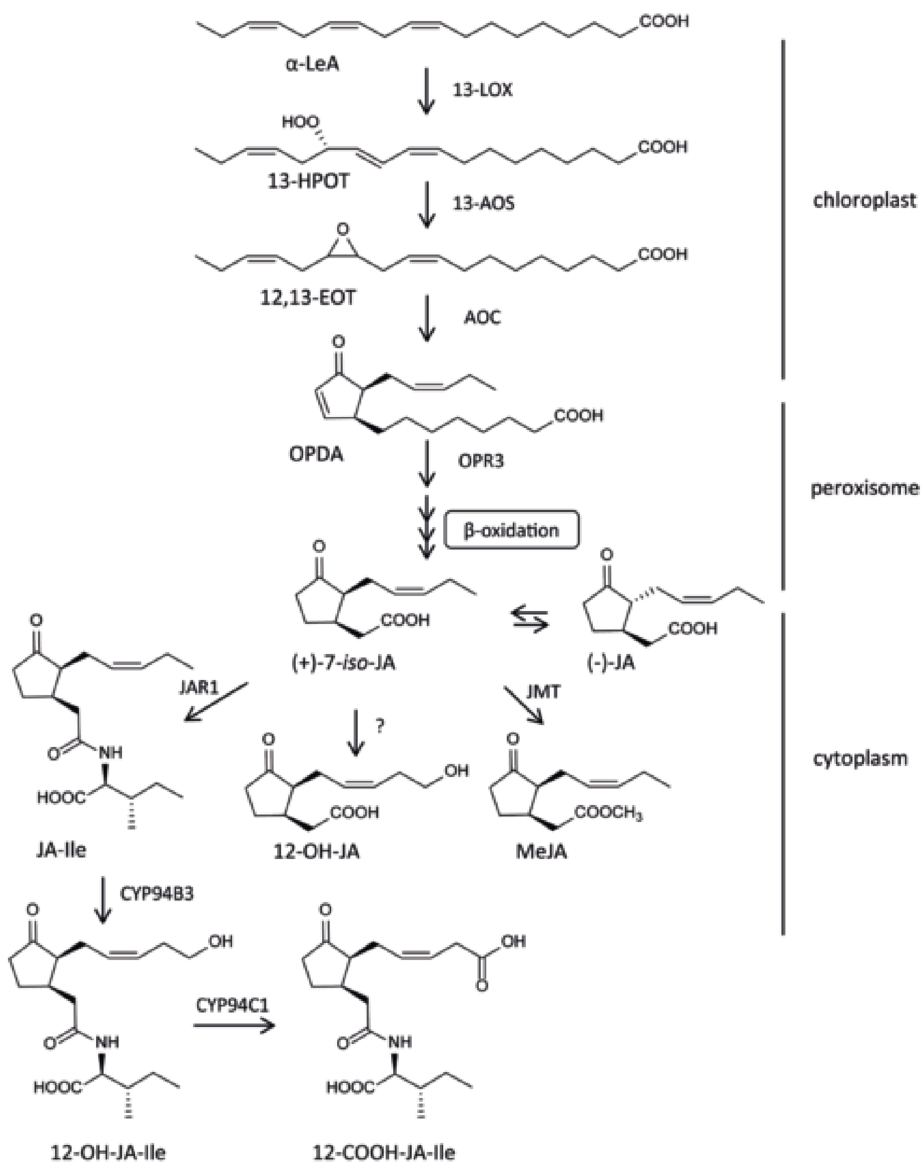

Figure S2. **Scheme of jasmonic acid biosynthesis pathway**

Adopted from Zhang (2016). 13-LOX, 13-lipoxygenase; 13-AOS, 13-allene oxide synthase; AOC, allene oxide cyclase; OPR3, OPDA reductase; JAR1, jasmonate resistant 1; JMP, Jasmonates carboxyl methyltransferase; α-LeA, α-linolenic acid; 13-HPOT, 13-hydroperoxylinolenic acid; 12,13-EOT, 12,13-epoxyoctadeca-trienoic acid; OPDA, 12-oxo-phytodienoic acid; (+)-7-iso-JA and (-)-JA for jasmonic acid; JA-Ile, jasmonoyl-isoleucine;12-OH-JA for 12-hydroxyjasmonic acid; MeJA, methyl jasmonate; 12-OH-JA-Ile and 12-COOH-JA-Ile for oxidized 12-hydroxy-JA-Ile.

CTGTTACCATCGGTGGCACTTTTCGGGCCTTTTCAAAGGAGAGCTTTCAGCAGCTTA
GGCAGCGAATTGAGGAGGTTATTGTTGGGCAAGCTGCTGTACAGAGATGCAATGCA
ACTGTGGATTTTTTAACAAAAGAAAAACCCTTCTTCCCTCCAACCGTGAACGATAAA
AACTTGCACAAACACTTCCAGAGAGTTGCAGGTGATATGCTTGGTAACGATCATGTA
AAAGACATGGAACCACTAATGGGATCGGAGGATTTTGCGTTTTACCAAGAGGTTATT
CCTGGTTACTTCTACCTACTCGGAATGCAGGATGAAACAAATGAAAAACTTGTTTCA
GTCCATTCACCTTATTTTAAAATCAACGAAGAAGCACTTCCTATCGGTGCTGCACTTC
AAGCATCTTTGGCTATCAGATATCTTCTCGAAGCACAACCACAAGTTCCTTCGT

Figure S3. ***NtJIH1*** **cDNA sequence used for RNAi construct**

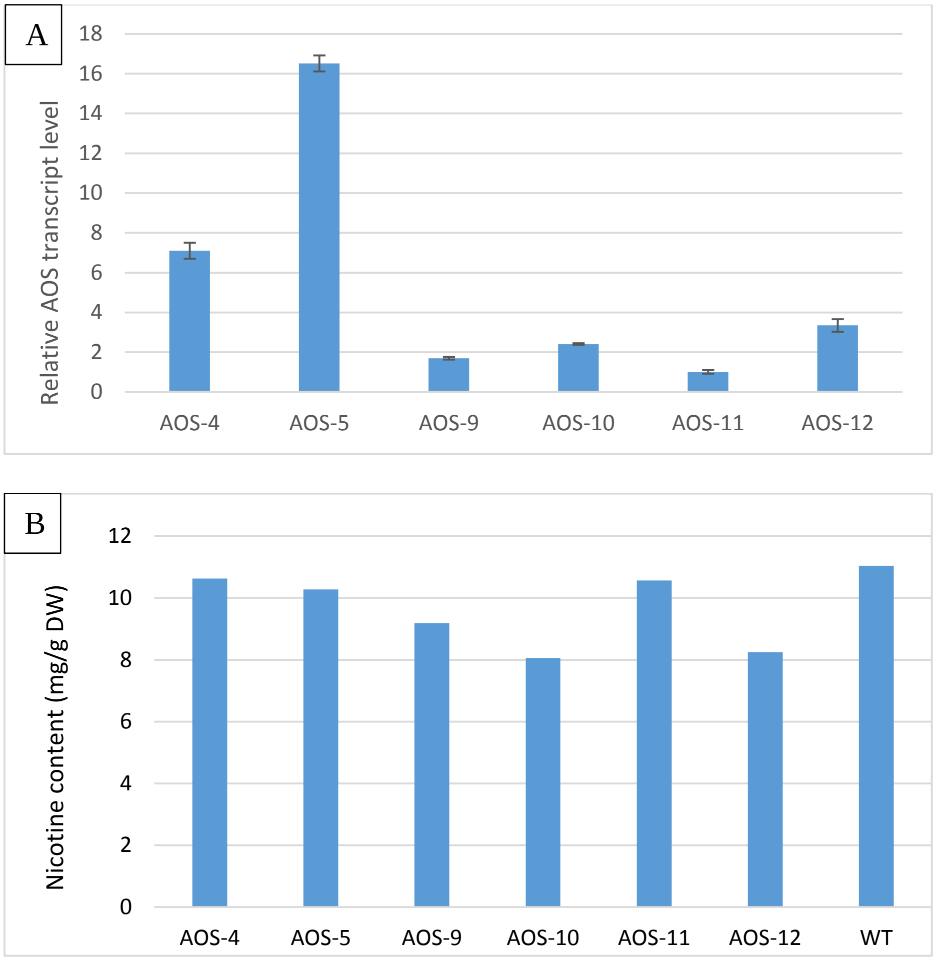

Figure S4. ***AOS*** **transcript levels and nicotine levels of AOS transgenic T_0 plants**

A. **Relative *AtAOS* transcript levels in AOS T_0 plants**. RNA was isolated from tobacco leaves collected before topping. Transcript level was determined by qRT-PCR. Values shown are means of 3 technical replicates. Error bar on the column represents standard error. Transcript levels were normalized to *actin* and displayed relative to the *AtAOS* transcript level of AOS-11 plant which is arbitrarily set at1.

B. **Nicotine content of AOS T_0 plants.** Seven days after topping, the top fully-expanded 12 leaves were collected, dried and ground. Total alkaloids were extracted from approximately 200 mg leaf tissues and individual alkaloids were quantified by HPLC-MS as described in the material and method section. The columns represent nicotine content (mg) on per gram dried leaf basis.

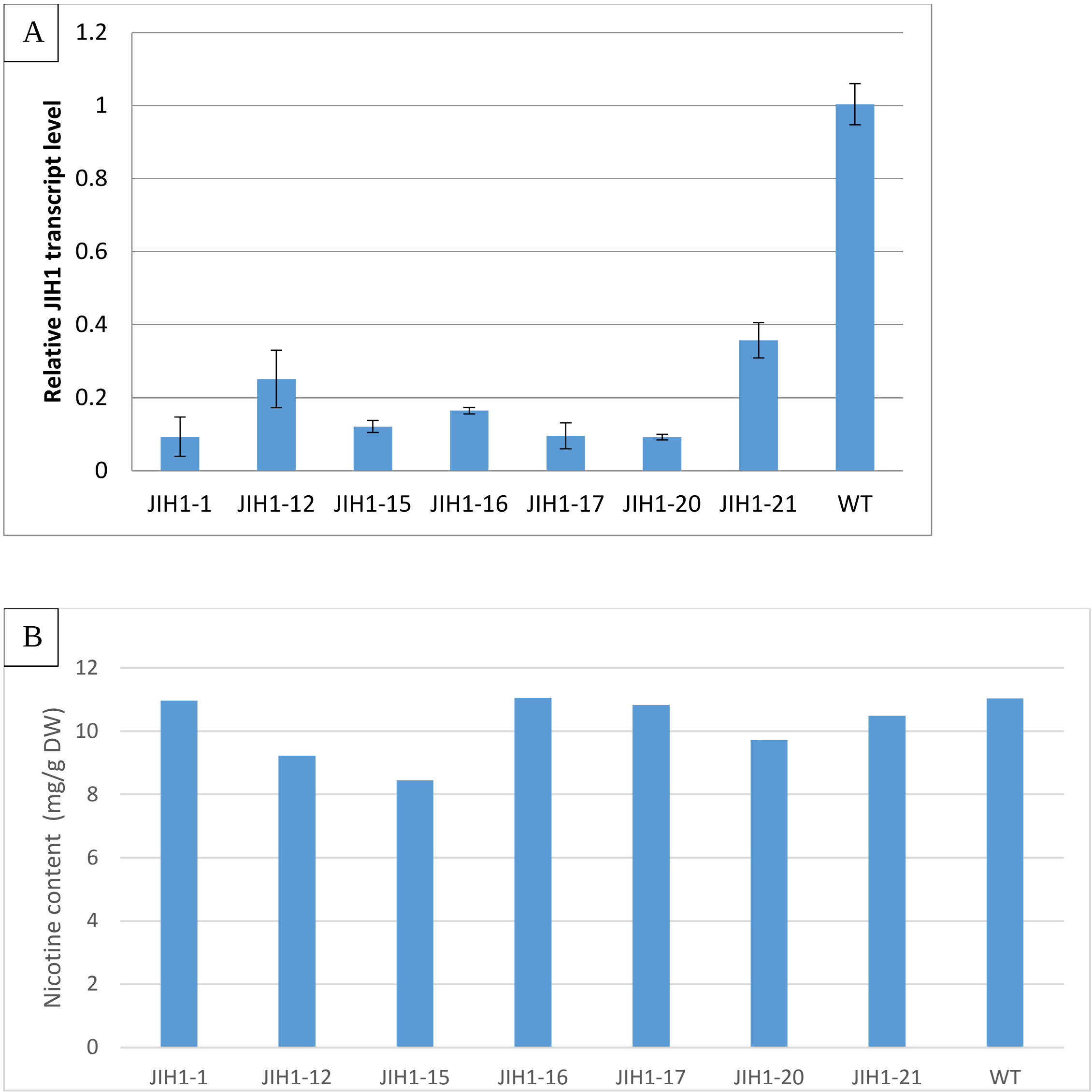

Figure S5. *NtJIH1* transcript level and nicotine content of JIH1 transgenic T_0 plants

A. **Relative *NtJIH1* transcript level in T_0 *JIH1* RNAi plants.** RNA was isolated from tobacco leaves collected before topping. *NtJIH1* transcripts were detected by qRT-PCR. Values shown are means from 3 technical replicates. Error bar on the column represents standard error. Transcript levels were normalized to actin and displayed relative to the transcript level of wild type which was arbitrarily set at 1.

B. **Nicotine content of *JIH1* RNAi T_0 plants.** Seven days after topping, the top twelve leaves were collected, dried and ground. Total alkaloids were extracted from approximately 200 mg leaf tissues and individual alkaloid was quantified as described in the material and method section. The columns represent nicotine content on per gram dried leaf basis.

Table S1. Primer sequences used to make gene constructs and to analyze gene expression. FP: Forward primer. RP: Reverse primer.

	Primers used for making overexpression constructs
AtAOS_FP	5’- GGACTCTAGAGGATCCATGGCTTCTATTTCAACCCCTT -3’
AtAOS_RP	5’- GACGGCCAGTGAATTCCTAAAAGCTAGCTTTCCTTAACGAC-3’
AtAOC2_FP	5’-GGACTCTAGAGGATCCATGGCTTCTTCAGCAGTGT -3’
AtAOC2_RP	5’-GACGGCCAGTGAATTCTTAGTTGGTATAGTTACTTATAACTCCGC -3’
AtJAR1_FP	5’-GGACTCTAGAGGATCCATGTTGGAGAAGGTTGAAACTTTCG-3’
AtJAR1_RP	5’- GACGGCCAGTGAATTCTCAAAACGCTGTGCTGAAGTAG-3’
AtOPR3_FP	5’-GGACTCTAGAGGATCCATGACGGCGGCACAAGG -3’
AtOPR3_RP	5’- GACGGCCAGTGAATTCTCAGAGGCGGGAAAAAGGAG -3’
AtLOX2_FP	5’- GGACTCTAGAGGATCCATGTATTGTAGAGAGTCCTTGTCGA -3’
AtLOX2_RP	5’-GACGGCCAGTGAATTCTCAAATAGAAATACTATAAGGAACACCCATT -3’

	Primers used to generate RNAi vector
JIH1-RNAi_FP	**5’-CAAATAATGATTTTATTTTGACTGATAGTGACCTGTTCGTTGCAAC AAATTGATGAGCAATGCTTTTTTATAATGCCAACTTTGTACAAAAAAGC AGGCTCTGTTACCATCGGTGGCACTTTTCG-3’**
JIH1-RNAi_FP	5’-CAAATAATGATTTTATTTTGACTGATAGTGACCTGTTCGTTGCAAC AAATTGATAAGCAATGCTTTCTTATAATGCCAACTTTGTACAAGAAA GCTGGGTACGAAGGAACTTGTGGTTGTGCTTC-3’

	Primers used for qRT-PCR
AtLOX2_FP	5’-TTTCTGGCCCGGGAAGTATG-3’
AtLOX2_RP	5’-AGCCAACCCCCTTTTGATGA-3’
AtAOS_FP	5’-CCGACGGTGGGGAATAAACA-3’
AtAOS_RP	5’-TAACGGAGCTTCCTAACGGC-3’
AtAOC2_FP	5’-ATATCGAAAACCCTAGACCAAGCAA-3’

AtAOC2_RP	5’-GAGATCTCCGAGACCGAACA-3’
AtJAR1_FP	5’-ACGGCTCATCAAGTCCAGAAACA-3’
AtJAR1_RP	5’-CAGGGTCAGTAGCGTTTCCA-3’
AtOPR3_FP	5’-AAACCCGGATTTGGTTTCGC-3’
AtOPR3_RP	5’-CCGTGTAGCCAACAACTGGA-3’
NtJIH1_FP	5’- GAAAAACCCTTCTTCCCTCCA-3’
NtJIH1_RP	5’- TCATCCTGCATTCCGAGTAGG-3’
NtPMT1_FP	5’-CGTGTAACCCTAGTTCTCGGA-3’
NtPMT1_RP	5’- GCTACTGCCTCAAAGAATGGC-3’
NtQPT2_FP	5’-AGAGGTGAAACCACCAGCAC-3’
NtQPT2_RP	5’-TCCCGTCTTCCTTTGCTAGA-3’
NtMYC2_FP	5’-GATGGGATGCTATGATTCGTATAC-3’
NtMYC2_RP	5’-CTGAAACACTAGCATGGTGCACATC-3’
NtA622_FP	5’-GGATGATAGAGGCAGAAGGA-3’
NtA622_RP	5’-TGACAACTTTGTCTCTAGGAG-3’
NtBBLa_FP	5’-CTGCTGATAATGTCGTTGATGCTC-3’
NtBBLa_RP	5’-CACCTCTGATTGCCCAAAACAC-3’
Ntactin_FP	5’- CTGAGGTCCTTTTCCAACCA-3’
Ntactin_RP	5’- GGCGACCACCTTAATCTTCA-3’

Table S2. Individual alkaloid levels of T_1 plants without topping.

Tobacco tissues were collected from the top 12 leaves in absence of topping. All the four types of alkaloids were quantified in the same HPLC procedure. Values shown in this tables are means of three replicate plants and standard errors. Unit: mg/g dry weight. Student's t test (α=0.05) suggested no significant difference between VC and any of the transgenic lines. VC: vector control.

	Nicotine	Nornicotine	Anabasine	Anatabine
VC	5.298 ± 1.013	0.138 ± 0.013	0.028 ± 0.003	0.206 ± 0.032
LOX2-20	5.688 ± 0.648	0.138 ±0.009	0.028 ± 0.002	0.214 ± 0.014
LOX2-33	5.826 ± 0.882	0.141 ± 0.012	0.033 ± 0.005	0.222 ± 0.066
AOC2-50	5.974 ± 0.058	0.142 ± 0.004	0.029 ± 0.001	0.209 ± 0.007
AOC2-49	6.431 ± 0.476	0.152 ± 0.006	0.030 ± 0.002	0.224 ± 0.005
JAR1-60	4.740 ± 0.532	0.185 ± 0.058	0.027 ± 0.002	0.218 ± 0.020
JAR1-20	4.678 ± 0.400	0.122 ± 0.006	0.029 ± 0.002	0.215 ± 0.017
OPR3-21	6.005 ± 0.903	0.138 ± 0.010	0.026 ± 0.0004	0.190 ± 0.006
OPR3-40	5.178 ± 0.178	0.132 ± 0.002	0.026 ± 0.001	0.168 ± 0.006
